# Re-surveying breeding forest bird communities in Western Oregon after 50 years: comparing 1968–1970 and 2020–2021

**DOI:** 10.3897/BDJ.13.e162862

**Published:** 2025-08-15

**Authors:** Nolan Michael Clements, W. Douglas Robinson

**Affiliations:** 1 Oregon State University, Corvallis, United States of America Oregon State University Corvallis United States of America

**Keywords:** breeding bird communities, benchmark survey, bird abundance, long-term change, Pacific Northwest

## Abstract

**Background:**

Accurate assessments of changes in breeding bird populations require regular, structured surveys or, alternatively, carefully documented benchmarks that can be precisely repeated. We re-surveyed a historic benchmark of forest bird communities in Western Oregon, USA, originally conducted by Stanley Anderson between 1968-1970. Anderson’s thesis uniquely preserves detailed plot locations, species density estimates, vegetation structure summaries and methodological descriptions — an uncommon level of documentation for the time. To facilitate accurate comparisons and future re-surveys, we explain how we aligned our methods with Anderson’s and incorporated modern bird counting techniques. We also provide our raw data, metadata and methodological details to ensure transparency and reproducibility.

**New information:**

Anderson’s work presents unusually old and detailed datasets of forest bird communities preserved from the Pacific Northwest, USA. It provides a unique opportunity to examine long-term changes in breeding bird communities within these forested landscapes affected by anthropogenic influence. The data and methods presented here are well-documented, ensuring that this benchmark can be effectively used for precisely repeatable re-surveys and comparative studies.

## Introduction

Changes over the last half century in forest cover, management practices and climate prompt continual monitoring of the bird communities of Western North America. Apparent declines of the avifauna in the conifer forests of this region emphasise this need (Phalan et al. 2019, Rosenberg et al. 2019). These and other assessments of changes over long time periods (> 30 years) in these bird populations critically depend on accurate and well-preserved data. Such data are particularly valuable in the Pacific Northwest, where a long history of anthropogenically driven forest dynamics offers opportunities to investigate avian responses to land-cover change. Many research efforts have focused on the impact of silvicultural treatments on avian communities in western Oregon (e.g. McGarigal and McComb (1995), Betts et al. (2010), Ellis and Betts (2011), Cahall et al. (2013), Rivers et al. (2019), Harris and Betts (2021)), but comparatively few have investigated changes in abundance. Those that have done so (Rosenberg et al. 2019, Phalan et al. 2019) have relied on data from the United States Geological Survey Breeding Bird Survey (BBS), but these surveys are restricted to main roads and fail to survey core habitats. Alternatively, other researchers interested in long-term population trends from areas without consistent monitoring have utilised information preserved in historical ornithological works (Igl and Johnson 2005, Tingley 2017, Ellis et al. 2019, Clements and Robinson 2024). These snapshots of avian communities provide benchmarks which can be re-surveyed, allowing for analysis of potential changes in abundance (Robinson 1999, Iknayan and Beissinger 2018, Clements and Robinson 2024, Clements et al. 2025), distribution (Tingley et al. 2009, Tingley and Beissinger 2009) and shifts in species composition (Tingley and Beissinger 2013, Curtis and Robinson 2015, Curtis et al. 2016). However, the level of detail preserved varies substantially amongst these historical datasets and this uncertainty demands creative methodological adjustments to ensure the quality of contemporary re-surveys and comparisons (Igl and Johnson 2005).

In this paper, we preserve details of our re-survey of the breeding bird communities in the central Oregon Coast Range, USA, originally surveyed in 1968-70 by Stanley H. Anderson (1939–2005). Under John A. Weins, Anderson’s M.S. and PhD work described fundamental aspects of western Oregon's avifauna, including abundance, seasonality, habitat associations and community structure (Anderson 1970b, Anderson 1972, Anderson 1970a). Anderson's academic life took him away from the Pacific Northwest to Kenyon College in Ohio, but his contributions as a graduate student to the understanding of the region's birds are cited in greater than 150 scientific publications as of 2025 (Gitzwiller 2006). In his doctoral thesis, Anderson presented details on bird counting methodology, plot locations and density estimates for each species – in the process, preserving a high quality benchmark. This is one of the only datasets in the Pacific Northwest to preserve these types of data. Our objectives here are to: 1) provide extensive details about how we interpreted and utilised information about Anderson’s original study area, methodology and data; 2) preserve our re-survey protocol and additional methods; and 3) make the data resulting from our re-survey freely available and well-supported by metadata.

## Sampling methods

### Study extent

Anderson selected 10 plots located on the eastern slope of the central Oregon Coast Range, all within 20 km of Corvallis, Oregon. These plots were quarter sections (each one quarter mile by one quarter mile or 64.8 ha) identified, based on the United States Public Land Survey System information (township, range, section and quarter section) and named in a numeric sequence (plots 1-10; Table 1). We selected seven of these 10 plots, because plots 1 and 2 were not primarily coniferous forest and plot 10 had been altered by forest harvest practices immediately prior to modern re-survey efforts. Four plots were located in Oregon State University’s McDonald-Dunn Research Forest (plots 3, 4, 5 and 8) and dominated by Douglas-fir (*Pseudotsugamenziesii*) and big leaf maple (*Acermacrophyllum*). The remaining three plots were in Woods Creek Watershed (plots 6, 7 and 9) and were composed of relatively even-aged Douglas-fir interspersed with western hemlock (*Tsugaheterophylla*). Reduced access to plots 6 and 7 due to COVID-19-related restrictions resulted in multiple counts that were conducted outside the boundaries of the quarter sections (within 250 m), but in the same habitats as those within the plots. These surveys were categorised as “Out” versus those conducted within boundaries, classified as "In". We found no consistent differences in the bird communities between the two categories so combined them for analyses (Clements and Robinson 2022, Clements and Robinson 2024).

### Sampling description

**Bird Surveys**

Anderson reported that he counted birds using four methods (strip census, strip map, point quarter and sample count methods), but concluded that the sample count method was the most accurate (Anderson 1970b, Anderson 1972). This method involved conducting ten 10-minute stationary point counts spaced at intervals of approximately 95 m along irregular transects. Anderson did not explicitly describe where these transects occurred, but we assumed that he followed roads and trails. The understorey in these plots is characterised by dense vegetation and travelling through on foot would likely disturb birds and bias surveys. During each survey, Anderson recorded each bird seen within 18 m and counted birds he heard only if he was able to visually confirm them within the count period; although some aspects of the data suggest that heard-only birds were sometimes counted (e.g. the presence of rarely seen, but frequently heard canopy species). Surveys were conducted starting one hour after sunrise once per week between early June and mid-July, 1968-1970. His original data and notes were not preserved, so his exact effort is unknown.

To ensure the data produced from our surveys was comparable to Anderson’s, we also conducted 10-minute stationary points spaced 100 m along roads and trails (Suppl. materials 1, 2). As Anderson did not specify where within each quarter section he surveyed, we surveyed the entire quarter section and assumed that, because the habitat was generally homogeneous across the entire area, our comparisons would be reliable. If a bird was heard within 18 m, we made an attempt to visually locate it, ensuring we were following Anderson’s protocol. Each plot was surveyed at least once per year (2020-2021) by a skilled observer (NMC and/or WDR), between dawn and five hours after sunrise on good weather days in June. We made no attempt to survey the same exact locations within quarter sections between years. This effort resulted in 304 individual surveys and 4,934 observations of 63 species.

Additionally, we incorporated modern counting methods using unlimited radius count areas, recording the distance to each bird with a laser range finder when possible, tracking time-interval detections for individuals and noting the detection types (calling, singing, visual and/or flyover; multiples were allowed). Distance measurements allow for the implementation of distance sampling, a density estimation method which accounts for imperfect detectability (Buckland et al. 2001). Time-interval tracking allows for removal modelling, to account for imperfect perceptibility (Farnsworth et al. 2002); these data were not collected as removal data. For this component, most counts were divided into two back-to-back 5-minute surveys, totalling 10 minutes and 10 one-minute detection intervals. Within each 5-minute section, each individual bird was given a detection-no detection history, based on whether it was detected in one or more of the five one-minute intervals. A 1 was assigned when the bird was detected (regardless of detection type) within that interval and a 0 was assigned when the bird was not detected. Exceptions were made for 1,512 records for which the detection histories included two intervals in a 10-minute count (two 5-minute detection intervals). We also recorded the cardinal direction in which each bird was detected (useful in combination with distance estimates for improving species distribution models; Shen et al. (2025)) and the latitude and longitude of each survey location using a hand-held GPS unit, typically Garmin etrex 10 (accurate to within 10 m).

**Landscape Cover Assessment**

To assess the potential impact of landscape cover on changes in bird densities, we estimated percentage cover of land-cover types for each plot in aerial photos from the late 1960s and Landsat satellite imagery from 2021 (Suppl. materials 3, 4). Percentage cover was estimated by placing grids over landscape imagery and estimating the total area of each cover type (clear cut, early, mid and late successional forest) as a percentage. For plot 4, a substantial harvest event (covering > 75% of the plot) occurred in 1970 immediately following the completion of Anderson’s bird surveys. No other imagery collected within 5 years exists for this area, so these data are not included. Details on vegetation structure data and methodology are preserved in Clements and Robinson (2022, 2024).

## Geographic coverage

### Description

We surveyed the avian communities at seven of Anderson's original 10 plots. Exact plot locations are preserved in Table 1.

## Taxonomic coverage

### Taxa included

**Table:** 

Rank	Scientific Name	Common Name
species	*Gaviaimmer*	Common Loon
species	*Cathartesaura*	Turkey Vulture
species	*Accipiteratricapillus*	American Goshawk
species	*Accipiterstriatus*	Sharp-shinned Hawk
species	*Buteojamaicensis*	Red-tailed Hawk
species	*Strixvaria*	Barred Owl
species	*Glaucidiumgnoma*	Northern Pygmy-Owl
species	*Calypteanna*	Anna's Hummingbird
species	*Selasphorusrufus*	Rufous Hummingbird
species	*Chaeturavauxi*	Vaux's Swift
species	*Dryocopuspileatus*	Pileated Woodpecker
species	*Leuconotopicusvillosus*	Hairy Woodpecker
species	*Picoidespubescens*	Downy Woodpecker
species	*Sphyrapicusruber*	Red-breasted Sapsucker
species	*Colaptesauratus*	Northern Flicker
species	*Contopuscooperi*	Olive-sided Flycatcher
species	*Contopussordidulus*	Western Wood-Pewee
species	*Empidonaxhammondii*	Hammond's Flycatcher
species	*Empidonaxdifficilis*	Wester Flycatcher
species	*Empidonaxtraillii*	Willow Flycatcher
species	*Vireocassinii*	Cassin's Vireo
species	*Vireohuttoni*	Hutton's Vireo
species	*Vireogilvus*	Warbling Vireo
species	*Cyanocittastelleri*	Steller's Jay
species	*Perisoreuscanadensis*	Canada Jay
species	*Corvusbrachyrhynchos*	American Crow
species	*Corvuscorax*	Common Raven
species	*Prognesubis*	Purple Martin
species	*Bombycillacedrorum*	Cedar Waxwing
species	*Strixoccidentalis*	Spotted Owl
species	*Poecilerufescens*	Chestnut-backed Chickadee
species	*Sittacanadensis*	Red-breasted Nuthatch
species	*Certhiaamericana*	Brown Creeper
species	*Troglodytespacificus*	Pacific Wren
species	*Troglodytesaedon*	Northern House Wren
species	*Regulussatrapa*	Golden-crowned Kinglet
species	*Sialiamexicana*	Western Bluebird
species	*Catharusustulatus*	Swainson's Thrush
species	*Catharusguttatus*	Hermit Thrush
species	*Turdusmigratorius*	American Robin
species	*Ixoreusnaevius*	Varied Thrush
species	*Coccothraustesvespertinus*	Evening Grosbeak
species	*Spinuspinus*	Pine Siskin
species	*Spinustristis*	American Goldfinch
species	*Haemorhouspurpureus*	Purple Finch
species	*Loxiacurvirostra*	Red Crossbill
species	*Juncohyemalis*	Dark-eyed Junco
species	*Zonotrichialeucophrys*	White-crowned Sparrow
species	*Melospizamelodia*	Song Sparrow
species	*Pipilomaculatus*	Spotted Towhee
species	*Passerinaamoena*	Lazuli Bunting
species	*Icteriavirens*	Yellow-breasted Chat
species	*Pirangaludoviciana*	Western Tanager
species	*Pheucticusmelanocephalus*	Black-headed Grosbeak
species	*Geothlypistolmiei*	MacGillivray's Warbler
species	*Cardellinapusilla*	Wilson's Warbler
species	*Leiothlypiscelata*	Orange-crowned Warbler
species	*Setophagaoccidentalis*	Hermit Warbler
species	*Setophaganigrescens*	Black-throated Gray Warbler
species	*Oreortyxpictus*	Mountain Quail
species	*Patagioenasfasciata*	Band-tailed Pigeon
species	*Zenaidamacroura*	Mourning Dove
species	*Meleagrisgallopavo*	Wild Turkey
species	*Chordeilesminor*	Common Nighthawk
species	*Glaucidiumcalifornicum*	Northern Pygmy-Owl

## Temporal coverage

**Data range:** 2020-6-08 – 2020-6-30; 2021-6-10 – 2021-6-27.

## Usage licence

### Usage licence

Creative Commons Public Domain Waiver (CC-Zero)

## Data resources

### Data package title

Survey of breeding bird communities and quanitification of landscape change in the Oregon Coast Range

### Resource link

https://doi.org/10.5061/dryad.w9ghx3g29

### Number of data sets

2

### Data set 1.

#### Data set name

Clements_Robinson_breedingbirdsurveys_OregonCoastRange.csv

#### Data format

.csv

#### Description

A total of 4,934 records of birds occurrences from the Oregon Coast Range. Data exist as one bird per record. In case where flocks or pairs were observed, direction, distance, cue and detection history are duplicated for the number of birds observed. This format supports distance sampling analyses (Buckland et al. 2001), as implemented in Clements et al. (2025). For analyses that cannot accommodate duplicate records, we recommend aggregating species occurrences by date, time, latitude and longitude. Data and metadata are preserved in Suppl. materials 1, 2.

**Data set 1.** 

Column label	Column description
English Name	Contains the English species name of each bird species following the American Ornithological Society 2024 species names (http://checklist.americanornithology.org). Chesser, R.T.; Billerman, S.M.; Burns, K.J.; Cicero, C.; Dunn, J.L.; Hernández-Baños, B.E.; Jiménez, R.A.; Johnson, O.; Kratter, A.W.; Mason, N.A.; et al. AOU Checklist of North and Middle American Birds Available online: https://checklist.americanornithology.org/taxa/ (accessed on 11 September 2024).
Scientific Name	Contains the scientific species name of each bird species following the American Ornithological Society 2024 species names (http://checklist.americanornithology.org). Chesser, R.T.; Billerman, S.M.; Burns, K.J.; Cicero, C.; Dunn, J.L.; Hernández-Baños, B.E.; Jiménez, R.A.; Johnson, O.; Kratter, A.W.; Mason, N.A.; et al. AOU Checklist of North and Middle American Birds Available online: https://checklist.americanornithology.org/taxa/ (accessed on 11 September 2024).
Direction	To help observers keep track of different individual birds detected within a survey, direction was noted, based on the location of each bird at its initial detection. East = e, West = w, North = n, South = s, North-northeast = nne, Northeast = ne, East-southeast = ese, Southeast = se, South-southwest = ssw, Southwest = sw, West-northwest = wnw, Northwest = nw, North-northwest = nnw.
Distance	Distance between the observer and each bird at its initial point of detection was estimated and measured, when possible, to the nearest 5 m up to 50 m, the nearest 10 m up to 100 m and the nearest 25 m at greater distances when possible. Detections within 20 m were noted to within 1 m if the bird was seen. A few records above 20 m were also measured more precisely if the bird was seen or if circumstances allowed more precise measurements, such as recognition a bird must be in a specific tree.
cfsvd	Detection cues. The cues by which each bird was detected are noted, including calls (c) , song (s), drumming by woodpeckers (d), visuals (v) and fly-over (f). More than one cue can be noted for any given bird.
interval1_1	Noted as 1 if a bird was detected and 0 if it was not during the first one-minute interval of a 5-min count.
interval1_2	Noted as 1 if a bird was detected and 0 if it was not during the second one-minute interval of a 5-min count.
interval1_3	Noted as 1 if a bird was detected and 0 if it was not during the third one-minute interval of a 5-min count.
interval1_4	Noted as 1 if a bird was detected and 0 if it was not during the fourth one-minute interval of a 5-min count.
interval1_5	Noted as 1 if a bird was detected and 0 if it was not during the fifth one-minute interval of a 5-min count.
Time	Start time of each count period in HH:MM format.
Lat	Latitude in decimal degrees. Measured to 10 m accuracy with hand-held GPS unit.
Long	Longitude in decimal degrees. Measured to 10 m accuracy with hand-held GPS unit.
Plot	Anderson plot within which individual surveys were located. Plots begin with the abbreviation AP (Anderson Plot) followed by a number between 3 and 9.
CountLoc	Count location. Indicates whether survey location was within the quarter-sections originally designated by Anderson as his plots or not. In = latitude and longitude coordinates place the survey location within his original plot. Out = latitude and longitude coordinates place the survey location outside his original plot, but within 250 m of its boundary and containing habitat that, when viewed on satellite imagery taken within a year of our surveys, had similar habitat to that in the nearby plot.
Date	YYYY-MM-DD on which each bird was detected.
obs	Observer who conducted the count. Nolan M. Clements W. Douglas Robinson.
notes	An additional notation indicating that 1512 records used two 5-min time intervals instead 5 x 1-min intervals in back-to-back 5-min counts is made.
Crediting data use	A reminder is entered: If using any record(s) from this dataset in electronic or other published works, including other datasets, the record(s) are to be fully cited as: XXXXXXXXXXXXXXXXXXXXXXX
RecordID	Unique numerical identifier for each observation.
interval5_1	Noted as 1 if a bird was detected and 0 if it was not during the first five-minute interval of a 10-min count.
interval5_2	Noted as 1 if a bird was detected and 0 if it was not during the second five-minute interval of a 10-min count.

### Data set 2.

#### Data set name

Clements_Robinson_landcover_OregonCoastRange.csv

#### Data format

.csv

#### Description

Quantitative descriptions of land-cover characteristics in the Oregon Coast Range. Data and metadata are preserved in Suppl. materials 3, 4.

**Data set 2.** 

Column label	Column description
Plot	Anderson plot within which landcover is classified. Plots begin with the abbreviation AP (Anderson Plot) followed by a number between 3 and 9.
Year	Year aerial imagery was collected as YYYY.
Cover_type	Type of cover present within the plot boundaries described as clearcut, early successional (early), mid-successional (mid) and late successional (late).
Percent_cover	Percentage of total plot area represented by the cover type.
Crediting data use	A reminder is entered: If using any record(s) from this dataset in electronic or other published works, including other datasets, the record(s) are to be fully cited as: XXXXXXXXXXXXXXXXXXXXXXX.

## Supplementary Material

49F91F13-6046-53EF-A731-66B7E7D86439 10.3897/BDJ.13.e162862.suppl1 Supplementary material 1 Clements_Robinson_summerbirdsurvey_OregonCoastRange Data type avian occurrences Brief description Occurrence data for breeding birds of the Oregon Coast Range. See associated README for metadata information. File: oo_1384433.csv https://binary.pensoft.net/file/1384433 Nolan M. Clements, W. Douglas Robinson

D87AD8E7-E4C2-5ED3-9D7F-689937254462 10.3897/BDJ.13.e162862.suppl2 Supplementary material 2 README_Clements_Robinson_summerbirdsurveys Data type README Brief description Metadata information for bird occurrence data. File: oo_1384434.txt https://binary.pensoft.net/file/1384434 Nolan M. Clements, W. Douglas Robinson

6F483703-B7FF-54E9-8E0D-D260247A14D7 10.3897/BDJ.13.e162862.suppl3 Supplementary material 3 Clements_Robinson_landcover_OregonCoastRange Data type land cover composition Brief description Land-cover composition for categorical forest age classes from 1970 and 2021 in plots in the Oregon Coast Range. See associated README for metadata information. File: oo_1354790.csv https://binary.pensoft.net/file/1354790 Nolan M. Clements, W. Douglas Robinson

32D3C9CC-8B5A-5127-AC0D-DEC2CE5C6650 10.3897/BDJ.13.e162862.suppl4 Supplementary material 4 README_Clements_Robinson_landcover Data type README Brief description Metadata information for land-cover composition dataset. File: oo_1349822.txt https://binary.pensoft.net/file/1349822 Nolan M. Clements, W. Douglas Robinson

## Figures and Tables

**Table 1.** Location of each plot by decimal-degrees latitude and longitude coordinates of each corner.

	Northwest corner		Northeast corner		Southwest corner		Southeast corner	
Plot	Latitude	Longitude	Latitude	Longitude	Latitude	Longitude	Latitude	Longitude
3	44.63582	-123.31238	44.63582	-123.30209	44.62921	-123.31238	44.62921	-123.30209
4	44.62921	-123.35327	44.62921	-123.34378	44.62118	-123.35327	44.62118	-123.34378
5	44.62921	-123.33279	44.62921	-123.32346	44.62118	-123.33279	44.62118	-123.32346
6	44.53446	-123.53619	44.53446	-123.52638	44.52732	-123.53619	44.52732	-123.52638
7	44.54191	-123.52638	44.54191	-123.51644	44.53446	-123.52638	44.53446	-123.51644
8	44.62921	-123.32346	44.62921	-123.31238	44.62118	-123.32346	44.62118	-123.31238
9	44.53446	-123.55616	44.53446	-123.54663	44.52732	-123.55616	44.52732	-123.54663
